# Digital patient-reported outcomes in inflammatory bowel disease routine clinical practice: the clinician perspective

**DOI:** 10.1186/s41687-022-00462-x

**Published:** 2022-05-19

**Authors:** Amalie Søgaard Nielsen, Charlotte W. Appel, Birgit Furstrand Larsen, Lisa Hanna, Lars Kayser

**Affiliations:** 1 Diagnostic Centre, University Research Clinic for Innovative Patient Pathways, Silkeborg Regional Hospital, Silkeborg, Denmark; 2 Department of Public Health, Section of Health Service Research, University of Copenhagen, Øster Farimagsgade 5, 1014 Copenhagen K, Denmark; 3 School of Health and Social Development, Deakin University, Geelong, Australia

**Keywords:** Digital patient reported outcomes, Digital health, eHealth

## Abstract

**Background:**

Use of digital health services, such as digital patient-reported outcomes, depends on many different human factors as well as digital design solutions. One factor is clinicians’ attitude towards the system, their reasoning behind the using system and their perceptions of patients’ ability to engage with digital health systems. This study aimed to explore hospital clinicians’ attitudes towards digital patient-reported outcomes used in the routine care and treatment of inflammatory bowel disease, and to explore the potential role of clinicians’ attitudes in influencing patients’ use of digital patient-reported outcomes.

**Results:**

Twelve clinicians using digital patient-reported outcome assessments in the care of inflammatory bowel disease were interviewed about their experiences of, and perspectives on, using this service. Most participants supported the use of digital patient-reported outcome assessments in the care of most patients. Participants reported that most patients found the digital solution easy to use. They perceived digital patient-reported outcomes to have three main purposes: *prioritising resources; improving patients’ quality of life;* and *improving quality of care.* The *patient-clinician relationship* was of great importance to participants*.* Participants varied in their intention to use digital PRO, as some viewed the system as a positive but optional add-on for patients, whilst others intended to use the system with all eligible patients.

**Conclusion:**

Clinicians’ general support of using digital patient-reported outcomes might facilitate their use among patients with inflammatory bowel disease. The participants saw benefits in doing so for patients, clinicians and the wider health service. Clinicians’ attitudes towards the use of digital PRO in the care of their patients may influence patients’ uptake of health service.

**Supplementary Information:**

The online version contains supplementary material available at 10.1186/s41687-022-00462-x.

## Background

The assessment of digital Patient Reported Outcomes (PROs) is being used increasingly for a range of purposes in chronic disease management, including care of Inflammatory Bowel Disease (IBD) [1–4]. PROs have been defined as “any report coming directly from the patient about a health condition and its treatment, without interpretation of the patients response by a clinician or anyone else” [5]. In general, digital PROs allow assessments by which health professionals collect health-related data from their patients, often between consultations, via online questionnaires completed from home. This approach aims to support treatment, care and disease management, and reduce avoidable face-to-face consultations between doctor and patient [6]. Within IBD, patients daily life experience of the disease and changes herein associates with the state of disease and possibly need of action, thereby being a suitable condition for digital PRO assessment [7, 8]. Use of digital PROs also has the potential to increase patient empowerment and improve healthcare [1, 2, 9]. However, some patients seem to benefit more from this approach than others, and some do not engage with digital PROs at all [10].

Patient engagement, adherence and use of digital health services depend on many different human factors as well as digital design solutions [11–13]. One factor is clinicians’ perceptions of patients’ ability to engage with health systems, which may affect patients’ attitudes towards and uptake of the system [13, 14] and have been found to be a barrier for use [15]. In addition, when digital PRO assessments are introduced as a new intervention in clinical practice, where the assessment constitutes a follow-up session, this changes daily workflow and adds tasks for clinicians which might affect their attitude towards new solutions [16]. Use, and reflections on use, of PROs in daily clinical practice affect the realisation of the intervention [13]. Implementation efforts, including training for clinicians in how to handle digital PROs, and the information provided by PROs, are needed [17], especially when collecting data on health-related quality of life [16, 18]. Such implementation efforts aim to make a positive impact on the attitude of the clinicians and to secure a well-established solution for clinicians and patients [19]. Clinicians’ reasoning and justification for using digital PRO assessments may affect patients’ attitude to and uptake of the service [19]. Therefore it is of interest to understand clinicians’ reasoning related to use of digital services to be able to understand its effect on uptake and patients’ acceptance of technology. This study aimed to explore hospital clinicians’ attitudes, including their reasoning and motivation towards digital PROs used in the routine care and treatment of IBD.

## Method

This study was part of a larger action research project [20] on patient-reported outcome concepts at an outpatient IBD clinic at Silkeborg Regional Hospital in Denmark [8]. This project has reported on patients’ perspectives on digital PRO [21]; this paper presents clinicians’ perspectives.

### Setting

AmbuFlex is a publically funded, hospital-based generic web-administered PRO-solution which is used for a wide range of chronic diseases, including IBD, in Central Region Denmark since 2016. At the outpatient clinic for IBD at Diagnostic Centre at Silkeborg Regional Hospital (DC) which provides care to around 850 patients with IBD. 77% of patients are enrolled in the AmbuFlex IBD specific subset; AmbuIBD [8]. AmbuIBD is applied to replace face-to-face clinical follow-up consultations with Medical Doctors (MD) or Registered Nurse (RN); to prepare for a face-to-face consultation with a MD or RN; thus supporting patients in preparing and clinicians to target the issues of the consultation; and to report symptom exacerbation between consultations. MDs at the hospital identify patients for whom AmbuIBD is clinically suitable and enrol those consenting into the AmbuFlex system. Usually a RN then introduce the patient to the digital PRO system.

Through the digital PRO system, a questionnaire is sent to patients every 6–12 months using a national secure email system with a link to the questionnaire. Time between questionnaires is decided and adjusted on an individual basis, or the patient can access the questionnaire from a webpage at any time if they experience changes in symptoms or feel a need for contact. AmbuIBD consists of 44 questions based on existing questionnaires covering (1) disease activity (the Harvey Bradshaw Index (HBI) [22] and the Simple Clinical Colitis Activity Index (SCCAI) [23]), (2) well-being (Short Health Scale [24] and the WHO-5 Well-being Index [25]), (3) fatique (the Inflammatory Bowel Disease Fatigue Self-assessment Scale (IBD-F) [26]), and (4) questions on sexual dysfunction, medicine adherence, side effects, and weight. In addition, patients are able to write comments or request a phone call from the clinic. The AmbuIBD questionnaire was linguistically validated by the AmbuFlex section development team. A pre-specified algorithm in AmbuFlex colors each answer due to a cut-off value (green, yellow, red) in the receiving end of the system. The answers and the colour codes from the questionnaire are reviewed by trained RNs who decides if a patient needs a face-to-face-consultation, a phone call for additional information or if no action is needed at the time. If any items have a “red answer” the patient will receive at least a phone call, unless they are expected on attendance in few days. The RNs might consult a MD before contacting the patient if needed. Patients receiving medical treatment at the clinic every six to eight weeks receive the questionnaire prior to their treatment.

### Recruitment and data collection

All clinicians involved in the use of AmbuIBD (n = 12) at the current time (excepting this article’s co-author BFL) were invited to participate in an individual in-depth interview. All accepted: 6 MDs and 6 RNs with IBD experience, who all were users of the AmbuFlex system. Two RNs had used it since its implementation in 2016, and had been a part of the development team, whilst others only had used it a few times. Qualitative, semi-structured, face-to-face interviews were conducted between October 1st and December 20th 2018. The first author ASN, who had no prior relationship with the participants and no clinical background, conducted the interviews. The semi-structured interview guide focussed on participants’ attitudes towards and motivation to use technology, reasoning surrounding its use, and clinicians’ experience of patients’ engagement towards healthcare technology. Follow-up questions, exploratory questions and interpretative questions were used [27]. Every interview was recorded and transcribed verbatim by the interviewer. Interviews ranged in duration from 18 to 30 min (Additional file 1).

### Data analysis

Data were analysed thematically by two of the authors, using Braun and Clarke’s (2006; 2020) six-step inductive approach. First, the first author [name of author blinded due to peer review process], read the data several times. Second, data were coded descriptively in NVivo12 Plus. Third, categories were established from the descriptive codes. Fourth, overall themes were created based on the categories while the original data were revisited to ensure agreement. Fifth, the first and fourth authors ASN & LH, agreed on themes. The last analytic step was the writing of this paper, conducted after the contextualising event described in the next paragraph [28].

### Confirmatory triangulation

A group discussion of the themes in a clinical context was facilitated in April 2020 (online due to the COVID-19 lock down in Denmark) with two RNs and one MD from the site [29]. The two RNs had participated in the interviews. The aim of the group discussion was to examine if the themes were recognizable and relevant for the participants [30]. As a means for discussion, a set of questions based on the themes was conducted [29]. The themes were confirmed by the participants at this event and are presented in this paper.

## Results

The data show clinicians’ support for the use of digital PRO assessments in their practice. Their reasoning revolved around five themes: *prioritising resources; improving quality of life; improving quality of care; patient-clinician relationship;* and *ease of use.*

### Prioritising resources

There was considerable variation in how participants perceived the purpose of the Ambuflex system. However, all participants identified that a key purpose was the reduction in face-to-face consultations facilitated by AmbuFlex, and not having to spend time on healthy patients:

“Both… you know, it should be a good thing for both us and the patients. For the patients not to come in here and say everything is fine, I’ll leave again, see you next year. To avoid that consultation. For both the patient and us. Just to see them when something is up.” (RN 4)

However, while several participants acknowledged the intention was for the system to support cost-effectiveness and resource prioritisation, some doubted whether the implementation of AmbuFlex had actually saved money. Participants reported RNs spending substantial extra time managing the digital PRO system. As an example, one RN participant stated that they sometimes had to provide IT-support for patients:

“There you really have to have some IT skills to sometimes even help patients to, what exactly is the problem […] Well, it's shatter-annoying. Well it can get me all the way up in the red zone that we have to spend so much time on it.” (RN 4)

All of the RNs, whose task it was to coordinate the care surrounding the digital PRO system, stressed that this role was time-consuming and required complex coordination, which was a new job function to them. In addition, they reported the digital PRO implementation redistributed care work towards RNs from MDs:

“You can say we see a few more of those yearly controls, where some of them might have been seen by a doctor before, where it is just us who handle them now ... um ... and only if there's anything extraordinary that we have the doctors in.” (RN 2)

This was backed up by the MDs, who felt they had lost some control over patient care:

“Then of course I have to depend on trusting the RNs who are set to keep an eye on it so that they do so as well. Of course they do that.” (MD 5)

In addition, the amount of time RNs spent on consulting patients had reduced:

“We do not have these nursing controls with one year, half and full year checks of Crohn’s and colitis, we see that virtually no longer. So it's much more administrative in some way. We sit behind the screen and follow up on it.” (RN 5)

Half of the participants mentioned that part of the purpose for implementing the digital PRO solution was to be innovative and modern. A MD stressed the innovative ‘spirit’ at the hospital and said:

“It fits well with the spirit of the hospital that you want to be an innovative hospital and try to … [laugh]… do things different than you necessarily used to do.” (MD 2)

### Improving quality of life

In the participants’ reasoning around the use of digital PROs, fewer physical consultations were perceived to benefit patients and enhance their quality of life. One perceived benefit was that patients could avoid taking a day off work to attend the clinic, as described in this quote from a MD:

“I do think it is a waste of their [the patients] time to take time off work to come in here and tell that everything is fine.” (MD 5)

In addition, some participants believed that the digital PROs could support patient education and enhance patients’ knowledge of their disease, which could improve patients’ quality of life and quality of care:

“I think someone can use it [the PRO solution]. Yes, and that is also, what I sometimes try to say to people, but you can use it a little yourself, to score yourself, [...] but if you think like there could be some symptoms… Well, then you can go in and answer […] Is it as usual, or is there something that starts to change a little?” (RN 2)

Another RN emphasised the benefits of patients answering the same questions repeatedly over time*:*

*“*Now they are asked about joint pain every time, so they can think; well then, but it is probably related to my bowel disease and also all these extra-intestinal symptoms we ask every time.” (RN 5)

Not all of the participants supported this interpretation and some doubted whether patients’ answering digital PRO measures had an effect on their (patients’) health literacy at all.

“I doubt it [AmbuFlex contributing to patients’ understanding of symptoms][…] If, in the long run it will enhance their health literacy, well… I just don’t know.” (MD 4)

Participants differed in their opinion about how well-educated patients were regarding their illness to begin with. As one MD said:

“They are pretty well trained in reality ... that is if they have listened and read it [information material on IBD provided by the hospital].” (MD 5)

Another MD emphasized the difference between patients:

“It's very different. Someone uh ... someone does [know a lot about their disease], but of course there are many who do not. So, I also think ... that it is ... a little bit dependent on ... I also believe that IBD patients are very different. Some are very engaged. And someone would rather ignore their illness.” (MD 1)

A RN talked about how surprising it was to discover patients’ lack of knowledge about their IBD:

“It's really such an a-ha experience for us, too, that what we think they know, they don't always know. So… and it comes… it can get very visible in such a questionnaire.” (RN 4)

### Improving quality of care

One of the reasons participants cited for the implementation of a digital PRO system was to improve quality of care in a range of ways. For example, digital PRO assessment was perceived to provide an opportunity for clinicians to receive more reliable, valid responses from patients and thereby support quality care delivery. Clinicians felt that when patients were able to answer the questions at home in their own time they were better able to consider more accurate responses and to figure out what was most important to them at that moment. Another aspect of the digital PROs that clinicians believed supported quality of care was their capacity to collect”real time” symptom registration as the patient had the opportunity to respond to the digital PRO measure at the time they were experiencing symptoms:

“The clever thing is, they [the patients] can respond to the questionnaire whenever they want, and then we see it straight away. And then we act upon it. A lot of them know that thing about, well, I should have called the doctor, and then they are number seven waiting in line and then they never get to it, and then all of a sudden 10 days went before they actually got to tell us that they had diarrhea again.” (MD 1)

Participants perceived that digital PRO measures could support clinicians’ preparation for consultations, which in turn enhanced the quality of care provided. All participants agreed that this functionality was important; one MD shared how he prepared for a consultation by using PRO data:

“So, when they come to the clinic for something… then you go and check before the patient arrives… if there are any red answers or something, then you can see; ok, you can dive into what you really have to talk about.” (MD 5)

Similarly, a RN reported that the PRO data helped her figure out whether or not to contact a MD in advance of for example intravenous treatment:

“That thing about, well do we need a doctor? Or does it look fine and are there some things you just have to take into account. You can better prepare before they [the patients] arrive. So it doesn't come as a surprise or what to say, how they have it. You get a sense of is everything here as it usually is or is there something new?” (RN 2)

However, not all of the participating MDs could find the time to check patients’ PRO responses before every consultation:

“*Sometimes I must admit, I don’t get… I don’t check the response.*” (MD 6).

One of the perceived benefits of being able to prepare the consultation based on the patient’s answers was that the consultation had the potential to be more focused on the issues that worried the patient. A MD shared how this could force the subject of relevance to appear earlier in the conversation:

“Because you can say that then you might lead the conversation into the relevant topics right away rather than spend ... a lot of time on ... trivialities from when they arrive. It's only when a patient is about to leave out the door that they are going to talk about what they really wanted to talk about.” (MD 5)

In helping clinicians prepare for a consultation with a patient, another benefit of using digital PROs was that they gave clinicians an overview of the patient’s responses over time, and during the consultation could support detection of improvement or decline in symptoms or health status. Participants perceived this functionality to be useful in ensuring high treatment quality. Some participants also mentioned that patients may also find it useful to be able to review their previous results. One MD stressed the difference between digital and paper based patient-reported outcomes, and the benefits of the digital solution:

“If you had a piece of paper, then you had to scroll around in it and in this way you have them, even with this overview, where they are just next to each other the last four times, where you can then see , they have different colors and such. It's much more manageable.” (MD 3)

As one of the main reasons given for using digital PROs was to enhance clinicians’ preparation for a consultation with a patient, it is reasonable to consider whether the nature of the consultation itself might change as a result of using digital PROs. One MD said that using digital PROs’turns’ the conversation during a consultation:

“And then I can just as well start from it, and then I can then take the routine things I would otherwise ask afterwards, so that you can just as well get the conversation turned in relation to what I did earlier.” (MD 5)

Likewise, a RN stated that using digital PROs meant that the initiative was given to the patient during the consultation:

“[The consultation] becomes more patient-controlled, because they have been at home and prepared for themselves, and what is happening to them is what we should address when they are at the clinic.” (RN 4)

Most participants agreed that new topics arose during a face-to-face consultation as a consequence of using digital PROs, commonly issues about fatigue and sexual dysfunction. As one MD said:

“Then there are some of the answers that go on to more broad things than what we have been used to asking about, ie there is something about ... the patients are asked if they have fatigue, and something like that, we have not so routinely asked, so you can sometimes get another side to the patients. And some also give answers about their sex life not working, nor do I think there are many of us who used to ask that for an ordinary consultation.” (MD 6)

Due to the increased visibility of some symptoms caused by the digital PROs use, some clinicians reflected on whether or not the clinic could act on every reported symptom or health-related issue. One RN perceived a need to act on items that were in the questionnaire:

“I have had a few around... where they have the answer about sexuality ... where I think that now we have ... so those who have sat with that form have chosen that question have to be there ... and when it is red , then I go into it.” (RN 1)

Another RN expressed doubt on whether or not to act:

“Yeah ... So there is someone who is flagging ... that is, getting red, only because of that answer, and then you sit a little there, hm ... I have to call only to ask about it, or what. Well, so you sit there…” (RN 6)

### Perceived ease of use

All the clinicians agreed that the digital PRO system itself was very easy to use in their practice. Problems occurred only when there were technical complications:

“But then again; I know their hotline number by heart... to AmbuFlex. So, it has ... that is, that is... The Ambuflex solution has had some challenges, and we think that it is since their updates that there is simply something that simply does not run well enough.” (RN 4)

The usability of the PRO system was not perceived to be a problem for the patients, but it was believed that some patients had difficulty locating the Ambuflex website. In addition, the wording of the questionnaire was perceived to be difficult for some patients:

“I have experienced someone where they sit and answer the questionnaire while receiving biological treatment, who actually has difficulty reading what it says. And it is such a person who is… who is probably dyslexic or… is close by, at least. Who has difficulty understanding the meaning of it, and there are some single words they simply cannot read.” (RN 5)

In addition, clinicians reported that some patients did not know how to use a computer:

“Someone says, but I have a bad feeling about computers, it has nothing to do with me, and I can't figure it out at all. They may well say that, right.” (MD 5)

Some participants reported that patients thought there were too many questions on the PRO questionnaire—especially those patients who were receiving biological therapy at the clinic:

“They think there are many questions. Those who benefit the most from it are the ones we have who have colitis who do not get any treatment. Because they do not have to come in here and they do not have to send stool samples, and they do not have to have blood samples taken. So they have it easier. But many of the others, I actually think, think they get a little more work out of it, but on the other hand they can also see what we need it for.” (RN 5)

and.

“I think there are many [patients] who say that there are many questions to answer. They also think that these are the same questions, ie… someone is sitting and doing it every other month, the ones who get the infusion [biological treatment], they answer every two months, I think a little that they think, maybe even a little, that they do it for our sake.” (RN 6)

One MD pointed to the fact that the questionnaire is too comprehensive:

“We ask for example about their sexual life, or ... we ask for ... their everyday life, it affects everyday life ... and how sick they feel ... so there ... it is a long questionnaire if you ask me. And I'm just thinking ... if you have one specific problem that you would like help with, then there are many other things you must have crossed off before you can ... maybe get in touch with us with what really matters to you.” (MD 2)

Conversely, another MD mentioned the familiarity of the questions makes it easy for the patients to answer:

“These are the same questions we ask. That's why IBD is such a… a good thing to do AmbuFlex… because the questionnaires are… so they are already made, that's what we always ask because we have learned to ask from them.” (MD 1)

Three RNs reported that not all patients found the digital PRO questions relevant.

### Patient-clinician relationship

Some of the participants mentioned that digital PROs affected the patient-clinician relationship, which influenced their attitudes towards the digital PRO system and their intention to use it in their clinical practice:

“In the past, we saw our patients a little more often than we do now. And, I would say, for patients who have a newly diagnosed inflammatory bowel disease, I sometimes miss a little that I get to know the patient before they are assigned to AmbuFlex. Earlier, we had them going for years, and knowing that when we just hear the name, we know that ok, he really only calls if there is something extremely wrong and others where you know that they may well call , although there has just been a splash of blood in the toilet bowl. So that… dense feeling of who your patients are, I think it may not be as equally… as close as it was before…” (MD 6)

A RN, who was responsible for reviewing patient’s responses, thought of this a little differently:

“But since they are so good at using the comment field, we still follow them, when we see their answers and follow their lives in that way, right?. So I actually do not think that they are moving further away, that is.” (RN 4)

Another RN with the same task reflected further on this:

“so now I just have a name, and I can see how they are, but I do not know who they are. I haven't met them. So I think it may be a disadvantage. But it is not certain that the patients think so. [Laugh] It is always nice to know your patients, so ... But then ... the question is whether we did it already, because ... it was not certain that it was me they saw each time. If they came to a one-year check, there could be five different RNs they were in, so ...” (RN 5)

The majority of participants mentioned that some patients felt safe in contact with the clinic and were doubtful about the change in contact that is introduced by the new solution. A MD highlighted doubt about the patients’ perception on interpersonal relationship and social context:

”Someone they might also think that ... it is very impersonal, they would like to have talked to ... with a doctor or and a RN and ... and then you sit there all alone in front of ... in front of their PC and ... well that's not the same care you get that way.” (MD 3)

Another MD expressed a similar experience with some patients appreciating the opportunity to have a yearly talk with a health care professional:

“Someone can say that, well I think it's nice to come in and get a talk about ... once a year, and get to know, well something new has happened in the development that can mean something to me.” (MD 5)

One RN understood this as a safety issue:

“someone thinks it is very nice that, even if everything goes well, you just come in and say everything is fine, and you just get to see each other in the eyes. But it's a little different…” (RN 2)

Most of the participants mentioned that patients always had the opportunity to call the clinic as they were used to:

“A few are a little worried about losing contact ... if they lose contact completely, we usually say, but they are still seen every three years, and they can always call us and they can always ... so we are still here.” (RN 3)

and:

“the patients still have to opportunity call in here. We have not, as such, removed anything from them. Besides controls where they are feeling well. That's how I think it is.” (MD 1)

There was a difference in opinion regarding whether or not the digital PRO solution is a supplementary offer or a standard procedure. One RN said:

“So, I also think it is important that it becomes an offer and not a requirement. There must be room for those who continue to feel most comfortable in actually coming in and being seen and heard such face-to-face. That still have to be a possibility.” (RN 3)

An experienced RN contradicted this:

“ I don't really present it as much as an offer, but I say that's how we do it in the future.” (RN 5)

## Discussion

This study explored IBD clinicians’ attitudes towards the use of a digitally administered PRO assessment solution in daily clinical practice. The participating clinicians’ attitude related to the objective of PRO and the reasoning behind the use of PRO. The digital PRO solution was perceived as a means by which patients could report symptoms and other health related issues from home (planned or not-planned) and could allow clinicians to assess whether their patients needed a face-to-face consultation. In this way, using digital PRO was perceived as a way of prioritising healthcare resources. In this study, the resource implications of implementing digital PRO were not investigated, and research suggests PRO implementation does not always lead to resource savings [16, 31]. However, workflow had changed and resources were prioritised differently than before the implementatio]n of digital PRO. The participants accepted digital PRO as a relevant way to prioritise resources, and that affected their attitude to the solution.

Secondly, digital PRO assessment was perceived to enhance clinicians’ awareness of their patients’ health status and its change over time prior to the patient attending a consultation, as well as facilitating the patient’s sharing of information about other aspects of their experience that could improve their clinicians’ quality of care delivery. These perceived benefits affected participants’ support for the digital PRO solution, which is also seen in similar studies [15, 32, 33]

Finally, digital PRO assessment was perceived to improve patients’ access to the clinic and enhance patients’ health literacy, therefore supporting their quality of life. Improved patient access to the clinical setting has been found in other studies of patient-initiated digital PROs [31, 33]. These outcomes were highly valued by the clinicians participating in this study. All of the perceived reasons for use of a digital PRO solution above are related to its impact on the patient-clinician relationship, and this study found that perceived ease of use of the PRO system, by patients and clinicians, was a fundamental condition underpinning successful implementation.

The digital PROs investigated in this study contains different question types, which were perceived differently by clinicians. For example, the questions on health status or simple intestinal symptoms reporting were regarded as useful in a more direct way than the questions on health-related quality of life. Prior research has shown that disease-specific symptoms reporting can be used as preparation for consultations and in screening to avoid an unnecessary consultation [9]. When participants considered non-disease-specific question items, they reported being unsure about how to respond to the patient-reported information. Clinicians were less confident in non-disease-specific areas, and some may not have felt adequately trained to respond appropriately, as reported elsewhere in the literature [34, 35]. Similarly, responding to non-disease-specific symptomatology may be perceived to detract from time spent on disease-specific responses. The perceived loss of time from the core task is often seen as a barrier in implementation of new technology in healthcare [13]. Non-disease-specific questions are often not handled as individual questions but more as an overall health status, that can serve as an introduction to the conversation during consultation, but not as a screening mechanism. Most of the participating clinicians in this study seemed to think that the usefulness of the PRO solution lay within the disease-specific health status and symptoms questions, as found in other studies [18, 34, 36]. Only a few participants mentioned the value of obtaining a more comprehensive view of the patient’s overall status, but the ability to better prepare for consultations was seen as a value concerning the clinicians’ work. At the same time, clinicians also perceived digital PROs to be valuable for the patient, as they offered the patient the opportunity to talk about what most concerns them. This reflects PRO data perceived as a means to prepare and inform the consultation, and is well established in other studies [37]. With the right amount of training clinicians seem to be able to benefit from the introduction of digital PROs in their clinical work and in their relations to the patients.

As mentioned, the influence of using digital PROs on the patient-clinician relationship was an important consideration for participants. If clinicians have concerns about not getting to know the patient as they used to, these might affect their willingness to enrol patients in a digital PRO solution especially in the early stage of the patient’s outpatient care. This is often seen in digital health solutions that aim for fewer face-to-face consultations [38]. Meeting the patient in person was important to many of the participants in this study, and for some, a PRO-based follow-up could be initiated only when a relationship was established, as been found in other studies [34]. On the other hand, the PRO questionnaire may lead to an even better understanding of the patient, because the digital PROs are informing the consultation and giving a more holistic view of the patient [37].

The perceived validity of PRO data is a fundamental concern for any use of digital PRO. In the current study, participants did not seem to doubt the validity of the data. Serious doubts concerning this validity has been reported in other studies [32]; these doubts may lead to PROs being perceived as less useful and this has been found to be a barrier to their use [15]. Data being collected in “real-time” and the fact that the disease-specific questions are the same as used at consultations helped the participants of our study to trust the validity of the data. This is especially important when collection of PRO data is seen as a means to replace consultations. The interviews revealed some divergent views on the optimal length and content of the PRO questionnaire and its relevance to the clinical population. Clinical relevance of questions and answers is central for clinicians to accept a PRO solution, because the clinicians have to act on the answers continuously [34, 36]. Some participants believed it to be a burden to some patients to answer many questions that sometimes are hard to understand; whilst others thought the patients should be familiar with the questions from face-to-face consultations. After a revision of the clinical procedure at the study setting (after the data collection for this research), patients receiving biological treatment will only receive the questionnaire every other time they come to the clinic. A scoping review of patients’ reasons for not using digital PRO solutions conducted by some of the authors of the current study found no association between length and duration of the PRO questionnaire and use [39]. However, the current study’s findings reflected clinicians’ worries about patients’ willingness to use the digital PRO solution, which in turn affected clinicians’ attitudes towards the solution. In future implementations of PROs it might be important, to ensure the clinicians’ acceptance, to balance the need for information with the amount and complexity of questions in the PRO questionnaire.

The participants did have some concerns with regard to usability of the digital PRO’s, but this was mostly limited to concerns for the few patients that had trouble using computers or simply preferred to meet the clinicians face-to-face once a year. The most frustration with the PRO solution amongst clinician participants in this study was among dedicated RNs who had experienced issues impacting organisational workload and distribution of work, which has also been found in a study of a similar PRO solution for patients with epilepsy [16]. RNs felt they had to provide IT-support to patients without proper training or time to do so, and they felt they were using a lot of time on screening the incoming questionnaires. While the MDs participating in our study only had to review patients’ digital PRO answers while preparing for their consultations, the dedicated RNs reported that the introduction of digital PRO had significantly changed their job description. Changes in workflow and workload have been found to be a factor for success or failure of implementing digital health interventions [38]. During implementation of digital PRO solutions it is therefore important to handle the new tasks for clinical staff to ensure clinicians’ acceptance.

Regardless of these changes, all of the dedicated RNs seemed very positive about the digital PRO system. They could see interrelated benefits for the patients, the hospital and themselves as clinicians. A central consideration for participants in this study was patients’ perspectives and experiences, and value for the patient was regarded as value for the clinician. As the MDs prescribed the solution and the RNs handled it, it was important that they supported it for the patients to be satisfied in using the solution. Clinicians (especially RNs) need to maintain a positive attitude towards digital PROs, even if some patients complain about a long and complex questionnaire. The intention to use digital PROs on all eligible patients was different amongst participants; some viewed the digital PROs as a positive add on for some patients, but not something all patients had to use, whilst others had the intention to use the solution with all eligible patients.

Clinicians’ use of the technology might act as a hurdle for patients’ use (if the clinician never asks, the patient will never get a chance to use the PRO solution), but the clinician’s attitude towards the technology can be a factor influencing patient use as well. In classic theories on technology acceptance, *perceived usefulness and perceived ease of use* are main indicators for acceptance [40]. As it is the clinician who explains the usefulness and the ease of use to the patient, the clinician’s attitude is an external variable that could affect patients’ perceived usefulness as well as the perceived ease of use. In this study, clinicians found digital PROs useful, especially as a means to enhance quality of care and prioritise resources, which meant they were likely to recommend digital PROs to their patients and thereby enhance the patients’ perception of the usefulness of digital PROs. Of course, other external variables will affect both the perceived usefulness and ease to the patient. Likewise, the patient’s attitude can be an external variable that affects their clinicians’ acceptance of the service in a feedback loop where the patient reports back to the clinician and thereby forms the clinician’s perception of the usefulness and ease of use of the solution. This inter-dependence was obvious in the findings from this study, as participants had their patients’ worries in mind and focussed substantially on the patient-clinician relationship.

### Strengths and limitations

The findings of this study were generated using a thematic approach and this may be influenced by the perspective of the researchers (and participants) and the questions and prompts used in data collection. The interview guide may have affected the results and enabled on eHealth Literacy and patient-clinician relationship in a way that might not have been as obvious with the use of other interview guides. However, the themes were validated by participants via the group discussion with the confirmatory purpose.

The clinicians’ perspective on the use of digital PROs in relation to the patients reflect their beliefs about their patients’ ability to use the solution, and their own first thoughts on the solution and its effect on their daily work. These beliefs and attitudes were shaped by the initial presentation of the PRO solution to clinicians. These two perspectives were not distinctively separated in the interviews, and reflected that the patients’ perspective is important to the participants, and that the patient-clinician relationship is of utmost importance.

This study collected participants’ accounts based on interviews and does not necessarily describe the actual use and work of the clinicians. However, the PRO solution is highly implemented and with close to 80% of the patients enrolled in the solution, all of the participants have some experience with the solution and are believed to use it.

This study was a single-site study and the results are not necessarily representative for other sites. In addition, this study examined the participants’ thoughts on the AmbuFlex system, and it may not generalize to other digital PRO assessment tools or other digital health services.

## Conclusion

The digital PROs used in the IBD clinic at Regional Hospital Silkeborg was perceived by clinicians to be useful as a tool to prevent avoidable face-to-face consultations, which benefitted patients, and to prioritise resources at the hospital. In addition, it was believed to have a possible positive effect on quality of life and quality of care. In this setting, the clinicians’ positive attitude towards the solution was believed to have a constructive effect on patient uptake. However, even though the participants had a high degree of acceptance of the solution, some were still reluctant to use the solution with all patients. It is important for clinicians to understand the needs and prerequisites of the individual patient when it comes to use of digital PROs. Specific training in the mechanisms behind use of digital PROs or screening of patients could accommodate clinicians’ concerns regarding patients that might have trouble using the solution.

## Supplementary Information

**Additional file 1:** Interview guide.

## Data Availability

Interview transcript is in Danish and kept at secure servers at University of Copenhagen for five years. Data is not freely available due to the privacy of participants, but parts of data may be released in anonymous form. Please contact corresponding author.
