# Supplementary material for: Digital patient-reported outcomes in inflammatory bowel disease routine clinical practice: the clinician perspective
Source: J Patient Rep Outcomes. 2022 May 19;6:52. doi: 10.1186/s41687-022-00462-x (PMC9117590; doi:10.1186/s41687-022-00462-x)
Supplement: Supplementary file 1 — Additional file 1: Interview guide. [file 41687_2022_462_MOESM1_ESM.docx]

| Domain | Questions (translated from Danish) |
| --- | --- |
| Experience with PRO | Tell me about your experience with AmbuFlex   - - How long have you been working with AmbuFlex? - - Do you know why AmbuFlex was implemented at the hospital? - - How were you trained in using the system |
| Ways of interacting with PRO | - What are your tasks in relation to AmbuFlex? - - Try to tell me how you typically work with AmbuFlex - please give examples - - Is there something difficult? In which way? - - Have your tasks changed due to AmbuFlex? |
| Motivation to use digital solutions | - How do you feel about using digital solutions in your work with the patient? - - What do you do to get the patient to use AmbuFlex? What if the patient stops using AmbuFlex? |
| Attitude | - What do you think of AmbuFlex in general? - - Is there anything that worries you about using IT in working with patients? - - Is there anything you think is particularly good about using IT in working with patients? |
| Patient experience | - How do you think patients experience AmbuFlex? - - What do you think they'll get out of it? - - What significance does AmbuFlex have for what you can offer the patient? |
| Engagement in Health (patient) | - Do you find that patients are generally very concerned about their own health? - - Do you find AmbuFlex changing anything about it? |
| Ability to actively engage with healthcare professionals (patient) | - Do you go through the answers with the patient during the consultation? - - Do patients expect you to have read their answers? - - Do you find that the patient asks other questions than before the use of AmbuFlex? |
| Attitude (patient) | - How do you think patients generally feel about using IT systems in their treatment? |
| Motivation to use digital solutions (patient) | - Do you feel that patients are comfortable using AmbuFlex from home? - - Do you find that patients are willing to use digital solutions in connection with their illness? |
| Health Literacy (patient) | - Do you find that patients generally have a good understanding of illness and health? - - Do you feel that AmbuFlex has changed something? Is it e.g. easier to talk about symptoms that were not previously perceived as part of the disease? - - Do you think AmbuFlex helps patients assess their own symptoms and health? |
|  | Anything else you want to add? |
|  |  |
